# Gastric bypass stent to reduce adverse events following endoscopic procedures in the descending duodenum: a conceptual proposal

**DOI:** 10.3389/fmed.2025.1555466

**Published:** 2025-07-10

**Authors:** Xiaomei Li, Conghua Song

**Affiliations:** ^1^Gastrointestinal Endoscopy Center, The Affiliated Hospital of Putian University, Putian, Fujian, China; ^2^Key Laboratory of Translational Tumor Medicine in Fujian Province, Putian University, Putian, Fujian, China

**Keywords:** gastric bypass stent, duodenum, endoscopic mucosal resection (EMR), endoscopic submucosal dissection (ESD), perforation, fistula

## Introduction

Endoscopic resection of mucosal and submucosal lesions in the descending duodenum has become increasingly common. However, it is frequently complicated by serious adverse events, particularly perforation and fistula, which can significantly threaten patient outcomes. The major clinical challenge lies in effective wound management following such procedures. In this article, we present a conceptual proposal that a promising application of the gastric bypass stent aimed at reducing adverse events after endoscopic resection in the descending duodenum.

## Conception

The gastric bypass stent, designed for obesity treatment, may provide a novel solution for protecting duodenal wounds. This device utilizes a highly biocompatible thin film to cover a segment of the duodenum, thereby redirecting food passage and minimizing direct contact between luminal contents and the intestinal mucosa. The high incidence of adverse events following duodenal endoscopic procedures is largely attributable to wound exposure to the complex intraluminal environment. We hypothesize that the gastric bypass stent can effectively shield the wound site, thereby reducing such complications. Theoretically, it offers an ideal physical barrier isolating the wound, while practically, its established clinical use and well-developed deployment techniques minimize procedural risks in this novel application. This concept thus holds promise to enhance the safety and efficacy of endoscopic surgeries in the descending duodenum, although further empirical investigation is required.

## Discussion

Endoscopic resection of mucosal or submucosal lesions in the descending duodenum has increasingly become a standard therapeutic approach for early neoplastic and premalignant lesions (1). However, this technique is frequently complicated by serious adverse events, most notably perforation and enteric fistula formation (2), which can significantly endanger patient survival. A major challenge remains effective wound management following resection in this anatomically complex region.

The gastric bypass stent (also known as the gastro-jejunal bypass sleeve or duodenal-jejunal bypass liner) (3), originally developed for obesity and metabolic disease treatment, offers a compelling alternative. This device employs a highly biocompatible thin film to cover a segment of the duodenum, effectively isolating it from exposure to luminal contents. Via minimally-invasive endoscopic procedure, this system reroutes food passage from the stomach so that it mixes with bile and pancreatic secretions distally, thereby reducing the direct contact between the duodenal mucosa and ingested food. Its minimally invasive nature and relative ease of deployment are notable advantages.

The high incidence of adverse events following duodenal endoscopy is primarily due to the wound surface being exposed to a complex intraluminal milieu comprising food particles, gastric acid, digestive enzymes, and bile acids. The gastric bypass stent appears uniquely suited to mitigate this risk by physically shielding the wound. We hypothesize that this stent could play a crucial role in protecting the wound surface after endoscopic procedures in the descending duodenum, thereby reducing the occurrence of these severe adverse events.

Regarding feasibility, two main considerations arise. Theoretically, the capacity of the gastric bypass stent to isolate a segment of the intestine with a biocompatible film provides an ideal method to separate the wound from the harsh intraluminal environment. This offers a strong rationale for reducing the risk associated with the wound-related complications. Practically, the gastric bypass stent is a well-established medical device with mature techniques ensuring its safe placement and remova, thus minimizing procedural uncertainties when repurposed for this novel indication.

Furthermore, unlike the long-term implantation required for obesity or metabolic indications, the stent in this context would be deployed only temporarily during the critical mucosal healing phase—generally 1 to 2 weeks. This shorter indwelling period is expected to reduce device-related complications such as stent migration, mucosal hyperplasia, or gastrointestinal obstruction. Prior clinical studies of duodenal-jejunal bypass liners for metabolic therapy have demonstrated acceptable safety profiles even with prolonged use (3). While preliminary theoretical rationale and analogous clinical evidence support this concept, its practical feasibility, safety, and generalizability must be confirmed through rigorously designed prospective clinical trials conducted across diverse clinical settings.

## Conclusion

The concept of using the gastric bypass stent to reduce adverse events following endoscopic procedures in the descending duodenum represents an innovative and potentially transformative approach. This strategy challenges the conventional management paradigms for post-endoscopic complications in this intractable anatomical region and opens a promising new avenue for investigation. However, comprehensive clinical studies are imperative to rigorously assess the feasibility, safety, and broader applicability of this approach before it can be adopted into routine clinical practice. Nevertheless, this concept holds significant potential to enhance the safety and efficacy of endoscopic interventions in the descending duodenum.
